# Establishment and Validation of a New Analysis Strategy for the Study of Plant Endophytic Microorganisms

**DOI:** 10.3390/ijms232214223

**Published:** 2022-11-17

**Authors:** Feng Chen, Xianjin Wang, Guiping Qiu, Haida Liu, Yingquan Tan, Beijiu Cheng, Guomin Han

**Affiliations:** 1 School of Life Sciences, Anhui Agricultural University, Hefei 230036, China; 2 National Engineering Laboratory of Crop Stress Resistance Breeding, Anhui Agricultural University, Hefei 230036, China

**Keywords:** endophytic microorganisms, 16S rDNA amplicon sequencing, plant transcriptome data, a new analysis strategy

## Abstract

Amplicon sequencing of bacterial or fungal marker sequences is currently the main method for the study of endophytic microorganisms in plants. However, it cannot obtain all types of microorganisms, including bacteria, fungi, protozoa, etc., in samples, nor compare the relative content between endophytic microorganisms and plants and between different types of endophytes. Therefore, it is necessary to develop a better analysis strategy for endophytic microorganism investigation. In this study, a new analysis strategy was developed to obtain endophytic microbiome information from plant transcriptome data. Results showed that the new strategy can obtain the composition of microbial communities and the relative content between plants and endophytic microorganisms, and between different types of endophytic microorganisms from the plant transcriptome data. Compared with the amplicon sequencing method, more endophytic microorganisms and relative content information can be obtained with the new strategy, which can greatly broaden the research scope and save the experimental cost. Furthermore, the advantages and effectiveness of the new strategy were verified with different analysis of the microbial composition, correlation analysis, inoculant content test, and repeatability test.

## 1. Introduction

Microorganisms are the most abundant and diverse biological resources on Earth [1], and endophytic microorganisms are commonly found in the roots [2], stems [3], leaves [4], flowers [5], fruits [6], seeds [7], and other tissues of plants. They can establish a relatively stable symbiotic and synergistic relationship with plants, and play a variety of roles in plants, such as nitrogen fixation, siderophore, stress resistance, and the promotion of phosphorus and potassium absorption [8,9,10,11]. For example, *Sphingomonas melonis*, an endophyte of rice seeds, can play an “extended immune system” role in the face of pathogen invasion, resulting in the failure of *Burkholderia plantarii* infection [12]. Thus, it is of theoretical and applied importance to carry out in-depth research on plant endophytes.

The main methods used to study endophytic microorganisms are culture and non-culture methods. The culture method is the traditional method of microbiological research, which is inexpensive and easy to master. However, due to the small number of media, a limited number of microorganisms can be cultured with the culture method [13], and the community structure obtained is often inaccurate. Based on modern molecular biology techniques and high-throughput sequencing technology, the non-culture method can analyze the composition of microbial communities with the gene sequences of microorganisms. The method overcomes the disadvantages of the culture method, which makes it difficult to carry out microbiological studies on a large scale, and has the advantage of processing a large amount of data at a relatively low cost [14].

At present, 16S/18S/ITS rDNA amplicon sequencing and metagenomic sequencing based on high-throughput sequencing technology have gradually become important methods for microbiome research, and are widely used in agriculture, industry, environment, food, and health [15,16,17]. High-throughput sequencing of marker genes is relatively low-cost and more widely used. However, the amplicon method can only amplify bacterial or fungal marker sequences in the samples to obtain the species composition. It cannot obtain the main microbial composition by one sequencing, and it cannot compare the relative composition information between bacteria and fungi, bacteria and hosts, fungi and hosts. Protozoa information, which has gained attention in recent years, is missed simultaneously. In addition, with the PCR technology, amplification deviation and difficulty in amplifying microorganisms with large amplification sequence differences exist, which can result in differences in the sequencing results, analysis results, and the actual situation [18,19,20,21]. There are many problems in the study of endophytic microorganisms in plants, and it is necessary to develop a better and lower-cost research method.

**Figure 1:**
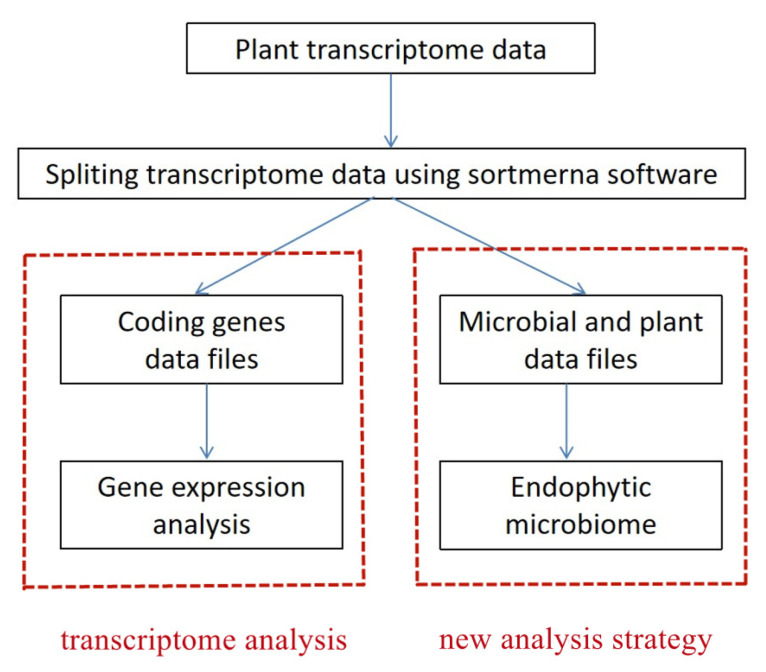
Schematic diagram of the new analysis strategy.

In this study, a new analysis strategy was developed to obtain endophytic microbiome information from plant transcriptome data. Based on the principle that plant transcriptome data also contain endophytic microbiome data, ribosome-coding sequences are distinguished from protein-coding sequences in transcriptome data, and the composition information of archaea, bacteria, fungi, viruses, protozoa, and other species with ribosomal coding sequences can be obtained (Figure 1). Compared with the analysis of microbial composition information by amplicon analysis or whole-genome sequencing of plant tissue, it can not only obtain the relative content information of various types of microorganisms, but also the relative content information of microorganisms and hosts with the new strategy. In addition, the existing transcriptome data can be used to obtain endophytic microbiome information, which can effectively save the cost of an investigation.

## 2. Results

### 2.1. Evaluation of rDNA Sequence Content in Raw Transcriptome Data

**Figure 2:**
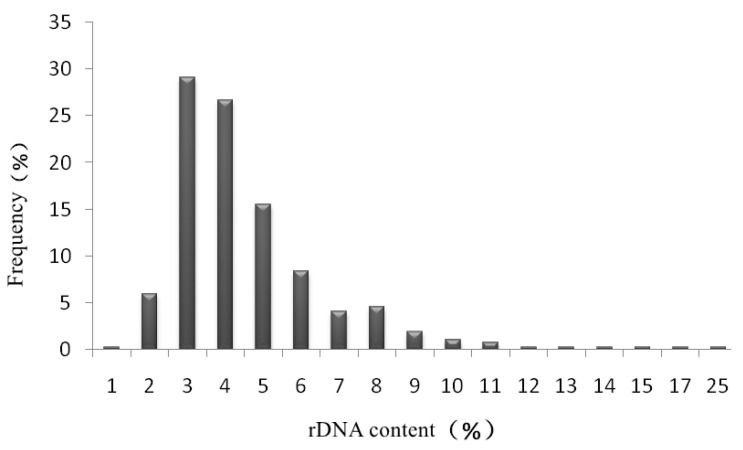
Assessment results of residual rDNA content in different transcriptome data. Note: the horizontal axis represents the rDNA content of the transcriptome data, and the vertical axis represents the proportion of different contents.

To investigate rDNA sequence content in raw transcriptome data, more than 400 transcriptome raw data, including maize, bean, and other plants, were downloaded from NCBI (Table S1). With the help of the SortMeRNA pipeline, the clean sequences were divided into rDNA-containing and rDNA-free sequences. The number of rDNA sequences in each transcriptome data ranged from 452,753 to 6,959,774 pairs with sequence content of 1.48% to 24.53%, and the sequence content of rDNA in most samples was 3% to 5% (Figure 2). The result clearly showed that although rRNA was removed as much as possible by the Oligo (dT) magnetic beads method or species-specific ribosome probe hybridization method before transcriptome sequencing, there were still some rRNAs that are not removed completely, and these residual rRNAs were also sequenced together with mRNAs by high-throughput sequencing. Since there are a large number of rDNA sequences in transcriptome data, these sequences can be used for further analysis to obtain information on species composition and relative content.

### 2.2. Analysis of the Species Composition with the New Analysis Strategy

**Figure 3:**
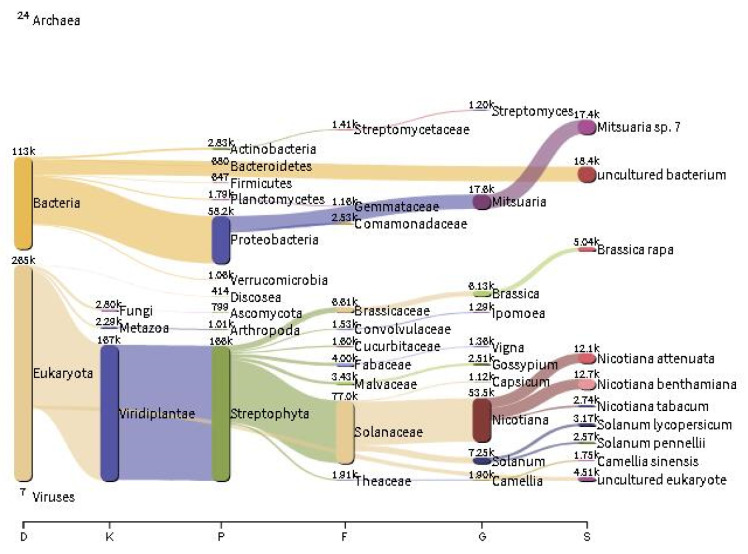
Sankey diagram of the species composition of tobacco roots. The contents of tobacco roots, bacteria, fungi, protozoa, archaea and viruses can be seen.

To compare the differences between the new analysis strategy and the amplicon sequencing method, some plant samples were collected and divided into two parts for each sample after surface disinfection. One copy was used for transcriptome sequencing, and the other copy was used for amplicon sequencing to compare the differences between the two methods. The transcriptome data were cleaned via Trimmomatic, and rDNA-containing sequences were obtained by using the SortMeRNA pipeline. Species composition information was retrieved via Kraken2. Results showed that the obtained species include genetic information on plants themselves, fungi, bacteria, protozoa, archaea, and viruses. Taking tobacco root as an example, it can be seen that most of the species’ information is tobacco, with smaller contents of archaea, bacteria, fungi, protozoa, and viruses in the Sankey diagram of species composition. The obtained microbiome was dominated by bacteria, with low levels of fungi, archaea, and viruses. Bacteria mainly included Proteobacteria, Actinomycetes, and Planctomycetes at the phylum level, and fungi mainly included Ascomycota at the phylum level (Figure 3).

### 2.3. Relative Microbial Nucleic Acid Contents

The relative nucleic acid content of the plant genome and microbiome in the obtained data can be calculated via Pavian. Results showed that the content of plant rDNA was above 96%, except for tobacco root, and the content of microbial rDNA was mostly lower than 4% (Table 1). The ability to obtain the relative content between plants and endophytic microorganisms is one of the advantages of the new strategy compared to the amplicon sequencing method.

The abundance of microbial species was counted and compared with Bracken software; it can be seen that bacteria and fungi were generally the most abundant, followed by protozoa and viruses being the least. In tobacco roots, the bacterial content accounted for 96.38% of all microorganisms, while in the leaves of Osmanthus fragrans, fungal content accounted for 79.26% of all microorganisms (Table 2).

### 2.4. Differences between the Results Obtained by the New Analysis Strategy and the Amplicon Sequencing Method

Six samples, including maize leaves, maize seeds, tobacco roots, tobacco stems, Osmanthus fragrans leaves, and Pittosporum leaves, were also analyzed with 16S rDNA amplicons, and the numbers of valid sequences were 67,132, 64,178, 60,034, 66,536, 65,988 and 64,695, respectively. The number of chloroplast sequences occupied a high proportion in each sample. After removing the chloroplast sequences, the numbers of effective microbial sequences obtained were 149, 17,590, 35,064, 6942, 1629, 6289, and 68, 236, 232, 65, 134, and 151 OTUs were annotated, respectively (Table 3).

**Figure 4:**
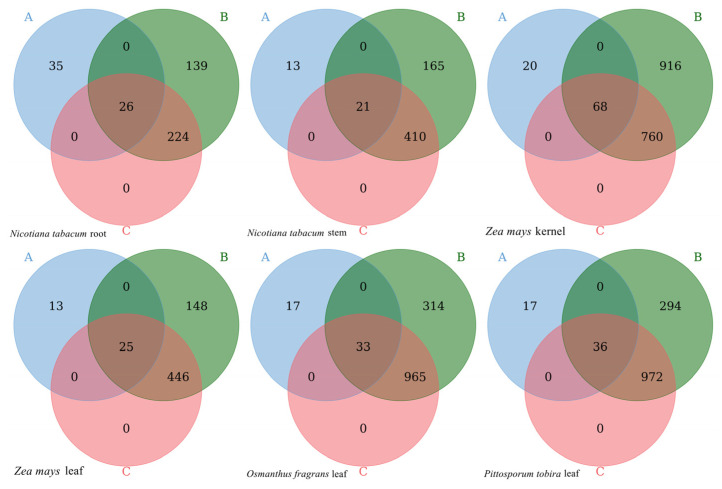
Venn diagram of the number of bacteria and microorganisms at the genus level with different methods. Note: A represents the number of bacterial genera obtained by the 16S amplicon sequencing method, B represents the number of all endophytic microbial genera obtained by the new analysis strategy, and C represents the number of bacterial genera obtained by the new analysis strategy. Venn diagram shows the distribution of the number of bacteria and microorganisms at the genus level with two methods, the overlapping parts represent common bacterial genus between A, B and C, while the non-overlapping parts represent unique bacterial genus.

To further reveal the differences between the results obtained by the new analysis strategy and the amplicon sequencing method, endophytic microorganisms in six samples were compared at the genus level to reflect the information about co-genera and unique genera with the two methods (Figure 4). Results showed that the number of species obtained by the two methods differed significantly, and the number of species obtained by the new strategy was much higher than that obtained by the amplicon sequencing method. The number of unique species accounted for 57.38%, 38.24%, 62.50%, 34.21%, 34.00%, and 32.08% of the species obtained by the amplicon sequencing method, while the number of shared species accounted for 42.62%, 61.76%, 37.50%, 65.79%, 66.00%, and 67.92%, respectively. In addition, the number of bacterial genera obtained by the new strategy accounted for 64.26%, 72.32%, 47.48%, 76.09%, 76.07%, and 77.42% of the number of genera of all species obtained, which indicated that there is a considerable proportion on archaea, fungi, viruses and protozoa genera by the new strategy. It can be seen that the endophytic bacteria obtained by the two methods have both commonalities and unique species. The new analysis strategy, using existing transcriptome data, can obtain part of the bacteria species with the amplicon sequencing method, but more other bacteria species and endophytes can be obtained with the new strategy than with the 16S rDNA amplicon sequencing method.

**Figure 5:**
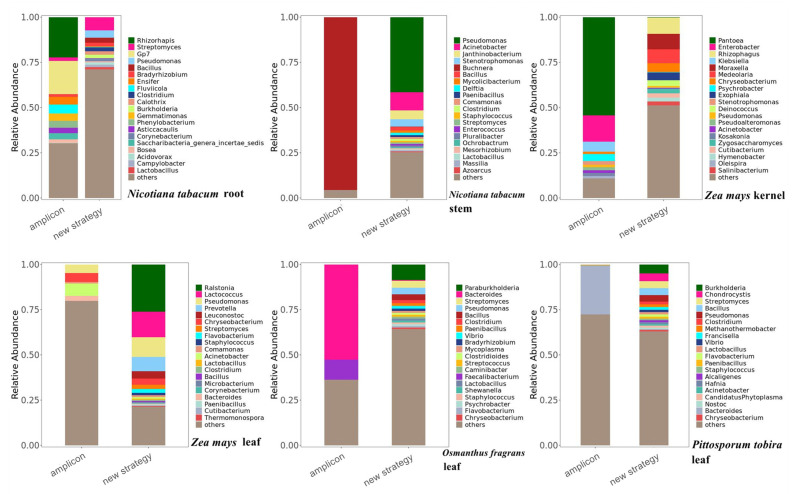
Bacterial community composition at the genus level with the two methods. The stack diagram shows that the abundance of bacteria obtained by the new analysis strategy is generally higher than that obtained by the amplicon sequencing method.

To identify the differences in the abundance of endophytic bacteria obtained by the two methods, the bacterial composition at the genus level was analyzed, respectively (Figure 5). The relative abundance of bacterial species obtained by the new analysis strategy is more balanced, while with the amplicon sequencing method, the relative abundance of one or several bacteria is often dominant, and the relative abundance of other bacteria is extremely low. For example, the relative abundance of Buchnera accounted for more than 90% of the bacterial community composition in tobacco stems obtained by the amplicon sequencing method, while other species were extremely low. The data obtained by the new analysis strategy might be more accurate in the bacterial community composition.

### 2.5. Correlation Analysis of Microbial Abundance between the Two Methods

**Figure 6:**
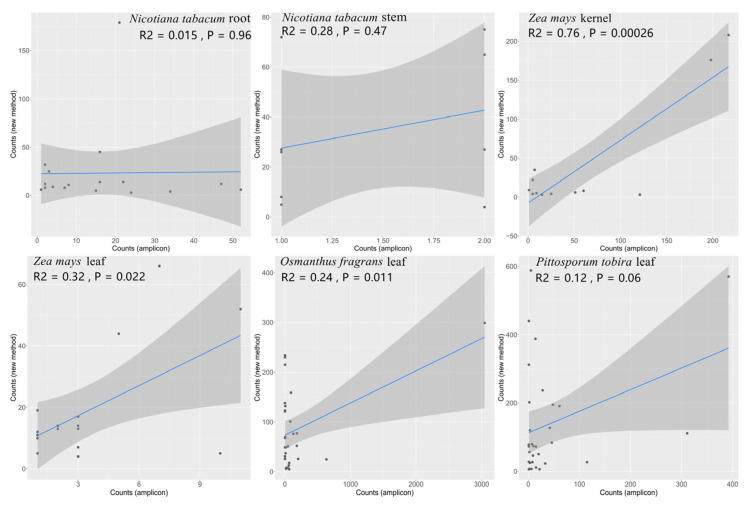
Scatter plot of the relevance of microbial information with different methods.

The correlation of the abundance of endophytic bacteria obtained by the two methods was analyzed. Results showed that each group of data has a linear positive correlation (Figure 6). The abundance of endophytic bacteria in maize kernel, maize leaves, and Osmanthus fragrans leaves, obtained by the two methods, was significantly correlated (*p* < 0.05), which showed that the data are consistent for both methods. There was no significant correlation in the abundance of endophytic bacteria in tobacco roots, tobacco stems, and Pittosporum leaves.

### 2.6. Reliability and Advantages of the New Analysis Strategy

To further examine the validity and reliability of the new analysis strategy, some transcriptome data of plants inoculated with microbes were selected to analyze the differences in microbial composition between the control and treatment groups. The transcriptome data of common bean inoculated with Xanthomonas, maize root inoculated with AM fungi, and maize seedlings inoculated with Ustilago were selected from the NCBI database. The endophytic microbial composition information was obtained from the transcriptome data of the control group and the treatment group, respectively, and the differences were analyzed. 

**Figure 7:**
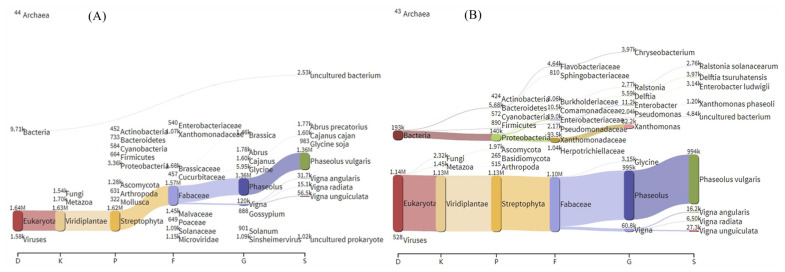
Sankey diagram of species composition of common soybean. (**A**) Species composition of common soybean before inoculation with *X. phaseoli pv. phaseoli* CFBP6546R. *X. phaseoli* cannot be seen in the picture. (**B**) Species composition of common soybean after inoculation with *X. phaseoli pv. phaseoli* CFBP6546R. *X. phaseoli* can be seen in the picture.

To observe the presence of inoculum in the treated and control groups, the species composition information before and after inoculation in the transcriptome data was inspected. Few corresponding inoculums were found in the control, while inoculum were found in common bean, maize root, Phaseolus vulgaris, and maize seedlings inoculated with Xanthomonas, AM fungus, Rhizobium tropici, Rhizophagus irregularis, and Ustilago maydis, respectively (Figure 7, Figures S1 and S2).

**Figure 8:**
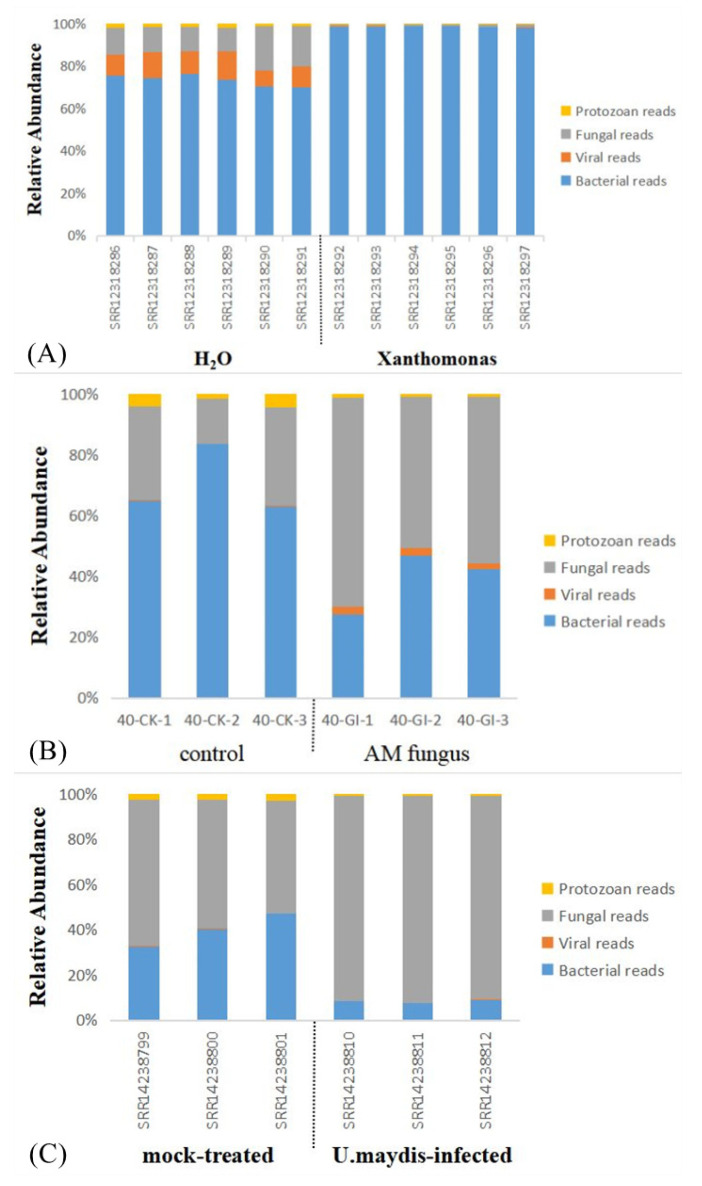
Differences in the microbial composition of plants before and after inoculation with microbes. Note: (**A**) Common soybean inoculation with *X. phaseoli pv. phaseoli* strain CFBP6546R. The content of bacteria in 6 samples inoculated with *X. phaseoli pv. phaseoli* strain CFBP6546R increased significantly compared with the 6 samples inoculated with H_2_O. (**B**) Maize root inoculated with AM fungus. The content of fungi in 3 samples inoculated with AM fungus decreased significantly compared with the control. (**C**) Maize seedlings inoculated with *U. maydis*. The content of fungi in 3 samples inoculated with *U. maydis* increased significantly compared with 3 mock-treated samples.

To investigate whether inoculation affected the composition of endophytic microorganisms, the composition of microbial species before and after inoculation was further investigated. The bacteria in the common bean control group were the majority, but fungi and viruses still occupied a considerable proportion. After inoculation with Xanthomonas, the bacterial content increased from 70.07~75.75% to more than 98%, while the fungi and viral content decreased from 7.21~21.07% to less than 1%. Before inoculation with AM fungi, the endophytic microorganisms in maize roots were mainly bacteria, while after inoculation with AM fungi, the bacterial content decreased from 62.99~83.52% to 27.49~42.43%, and the fungi content increased from 14.75~32.46% to 49.73~68.58%, becoming the predominant endophytic microorganism. In susceptible maize, the fungal content ranged from 49.77% to 64.73% before inoculation with U. maydis and increased above 90% after inoculation (Figure 8). Results showed that the relative content, varied among bacteria, fungi, protozoa, and viruses, can be obtained with the new analysis strategy. 

**Figure 9:**
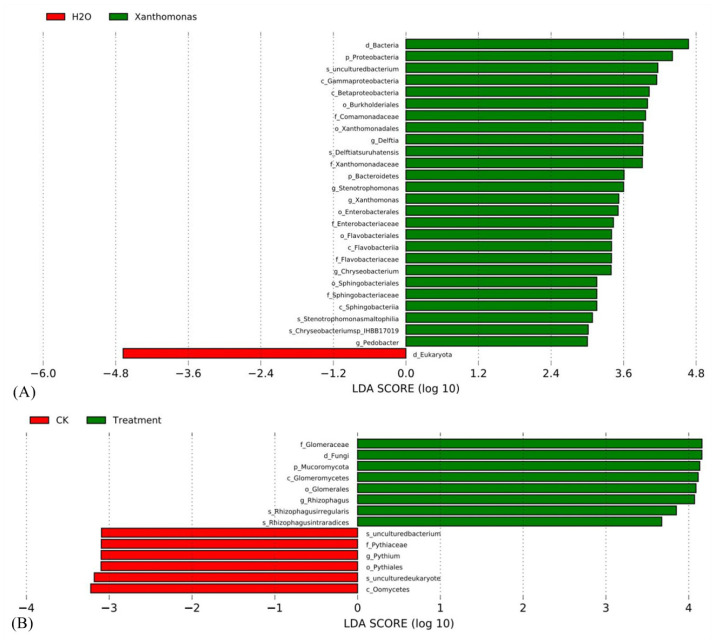
Biomarkers with a significant difference. Note: (**A**) Common soybean inoculation with *X. phaseoli*. H_2_O, the control sample of common bean; *Xanthomonas*, the common bean sample inoculated with *X. phaseoli pv. phaseoli* strain CFBP6546R. As the inoculum, *Xanthomonadales* at the order level, *Xanthomonadaceae* at the family level, *Xanthomonas* at the genus level can be seen as biomarkers after inoculation with *X. phaseoli*. (**B**) Maize roots inoculation with AM fungus. CK, the control sample of maize; Treatment, the maize roots inoculated with *R. irregularis* DAOM-197198. As the inoculum, *Rhizophagus* at the genus level, *Rhizophagus irregularis* at the species level can be seen as biomarkers after inoculation with *R. irregularis* DAOM-197198.

To examine the significantly different species of the above plants before and after inoculation with microorganisms, a LEFSe analysis on microbial species was performed to detect whether the inoculum is a biomarker with a significant difference after inoculation. The inoculated Xanthomonas and AM fungi were significantly enriched in the plants after inoculation and became the biomarkers with significant differences, which indicated that changes in composition and content can be detected accurately with the new analysis strategy (Figure 9).

**Figure 10:**
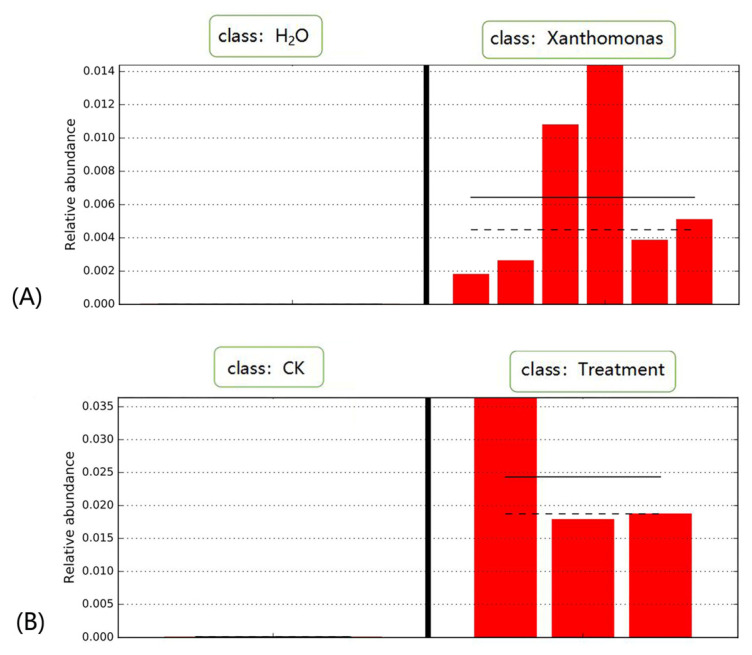
Relative content changes of plants inoculated microorganisms. (**A**) Common soybean inoculation with *X.phaseoli* strain CFBP6546R. Compared to the 6 samples inoculated with H_2_O, the content of *X.phaseoli* in 6 samples, inoculated with *X.phaseoli* strain CFBP6546R, increases significantly (*p* < 0.01). (**B**) Maize roots inoculation with *R.irregularis.* Compared to the 3 control samples, the content of *R.irregularis* in 3 samples, inoculated with *R. irregularis* DAOM-197198, increases significantly (*p* < 0.01).

The differences in the nucleic acid content of inoculated microorganisms before and after inoculation were further analyzed (Figure 10). In the common bean control group, the Xanthomonas content ranged from 0.00% to 0.0019% in all samples, with an average of 0.00073%. After 2 days of inoculation, the content of Xanthomonas ranged from 0.2175% to 1.3832%, with an average of 0.6729%. Compared to the control, statistical significance analysis showed a significant increase in Xanthomonas content in the treated group inoculated with Xanthomonas (*p* < 0.01). In the maize root control group, the AM fungi content ranged from 0.0134% to 0.0156%, with an average of 0.0144%. After 40 days of inoculation with AM fungi, the content of AM fungi ranged from 1.9251% to 3.7162%, with an average of 2.4882%. Compared to the control group, statistical significance analysis showed a significant increase in AM fungi content in the treatment group (*p* < 0.01). The results strongly demonstrated that microbial composition information can be obtained with the new analysis strategy. 

**Figure 11:**
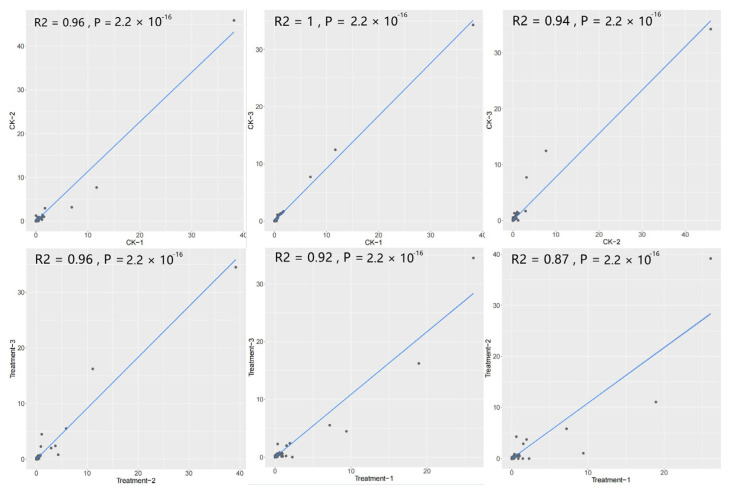
Scatter plot of the different biological repeats with new analysis strategy.

To examine the data stability and consistency of the new analysis strategy in this study, the samples with biological replicates from the inoculum experiments were used for investigation. In this study, correlation analysis was performed with replicate data from the transcriptome of maize roots before and after inoculation with AM fungus. The results showed a strong correlation in each replicate of the control and treatment groups; the correlation coefficient ranged from 0.87 to 1, with an average of 0.94. Statistical significance analysis showed that they were all significantly correlated (*p* < 0.01), which indicated that the new analysis strategy has strong data stability and consistency in mining endophytic microbial information from plant transcriptome data (Figure 11).

## 3. Discussion

Plants provide habitats for numerous endophytic bacteria, fungi, archaea, protozoa, and viruses, which is an important object of biological research [22,23,24]. At present, the research methods for endophytic microorganisms are mainly based on isolation and culture, and the amplicon sequencing method. In recent years, plant tissues were ground and the total DNA was extracted for resequencing in some studies [25]; certain types of microorganisms of interest can be analyzed with an online server. Although microbial composition information can be obtained by genome resequencing of plants, the dominant plant genome sequence is wasted. Since the endophytic microbial information can be obtained by plant genome resequencing, the expressed microbial gene also exists in the plant transcriptome data, which is the theoretical basis for the new analysis strategy in this study. About 1.5~24% of ribosomal gene fragments, such that the number of sequences exceeds 450,000 pairs, exist in a large amount of evaluated plant transcriptome data. The existence of a large number of ribosomal gene fragments has laid the foundation for the study of various biological compositions in plants.

In the study of mining maize endophytic microbes with resequencing, maize genome resequencing data were directly submitted to the online server MG-RAST for analysis [26]. At least 20 analysis methods have been reported for parsing metagenomic data, and different methods vary greatly in terms of server resource requirements, running speed, and database [27,28,29]. QIIME2 [30], mothur [31], and usearch [32] are the commonly used software for amplicon analysis in the research, while Kraken2 [33], metaphlan2 [34], and metaphlan3 [35] are commonly used in metagenome analysis. The data obtained from the transcriptome are short and cannot be used directly in amplicon processes. In addition, the amplicon analysis either only uses 16S rRNA databases of bacteria, such as Greengene [36], Silva [37], RDP [38], or the Unite database [39], only for ITS-amplified regions of fungi, while the transcriptome data include information on bacteria, fungi, protozoa, and viruses at the same time, so the existing amplicon analysis process cannot be used to analyze the rDNA sequence obtained from the transcriptome data. The comparison database of metaphlan2 and metaphlan3, which is the bacterial metagenome analysis process, is constructed with the protein-coding region sequences of bacteria, a small number of fungi, and viruses [40]; other non-protein-coding sequences are directly ignored, so the obtained rDNA sequences cannot be used for the metaphlan2 and metaphlan3 analysis processes. In contrast, Kraken2, the metagenomic analysis tool, not only runs extremely fast, but also uses both protein-coding and non-coding sequences. With the above analysis, Kraken2 is the most suitable software which can parse the obtained rDNA sequence files into species composition information, including plants and microorganisms. Combined with Kraken2 software, Bracken was used for the relative content analysis of different organisms. 

It can be seen from the 16S rDNA amplicon sequencing results of 6 samples that most of them were plant chloroplast sequences, accounting for 90.26%, 97.31%, 89.14%, 41.55%, 72.01%, and 99.41%, respectively, while microbial sequences only accounted for 9.72%, 2.46%, 10.38%, 58.35%, 27.19% and 0.22%, respectively, which resulted in few sequences that could be used for further analysis of endophytic microorganisms. This is due to the high similarity of the chloroplast and bacterial 16S rDNA amplified fragment sequences. The theory of intrachloroplast symbiotic origin [41,42] suggested that chloroplasts were originally an independently living cyanobacterium. When it was phagocytosed by eukaryotes, it performed photosynthesis for the host cell, while the host cell provided other living conditions for it. During the long-term symbiosis, chloroplasts were formed through evolution. Chloroplasts are more closely related to cyanobacteria than to anything else in plant cells. Chloroplasts are genetically independent of their own nuclear DNA, but have significant similarities with the bacterial genome. There are four rRNAs in chloroplast ribosomes (20S, 16S, 4.5S and 5S rDNA), so the chloroplast can be amplified when amplified with 16S rDNA universal primers. In addition, the numbers of chloroplasts in different organs of the same plant are different. For example, the number of chloroplasts is higher in leaves, where photosynthesis is required, while that is lower in roots where photosynthesis is not required, which also results in a higher proportion of chloroplast sequences in the 16S rDNA amplification results of plant leaves [43]. It can be seen that there are too many chloroplast sequences in the results with the 16S rDNA amplicon sequencing method, which leads to low efficiency and inaccuracy. The new analysis strategy can completely avoid this problem, which is also one of the advantages of the new strategy.

Comparing the results between the two methods, it can be seen that they can obtain partially identical results, and a larger variety and a large number of other bacteria can be obtained with the new analysis strategy. In terms of the number of bacterial species obtained, the new strategy is significantly better than the amplicon sequencing method, which showed a great technical advantage. Correlation analysis of the shared species obtained by the two methods showed that the relative contents of bacteria obtained by the two methods were positively correlated in the tested samples, while the other part of the samples lacked correlation. The reasons for the incomplete correlation may be the following: (1) The degenerate primers used in the amplicon to amplify different types of bacteria had certain selectivity and bias, and some bacteria may not have been amplified at all, resulting in biased results. (2) The database and abundance calculation algorithms used in different analysis processes were different. In the amplicon analysis, only the bacterial 16S rRNA database was used, and the results of the analysis pipeline contained some known and unknown OTUs, while the database used by Kraken2 was rich, covering almost all sequenced genomes, including a large number of bacteria, fungi, protozoan, plant, virus genomes, and the NCBI nt database. It is important to mention that only species with sequenced genomes could be detected via Kraken2. Different databases and analysis strategies may result in more species being obtained by the new analysis strategy, or a small number of species only identified with the amplicon method, which is the main reason for the lack of comparability between the results obtained by different analysis methods. The differences in microbial composition analyzed by different methods are waiting to be resolved by future algorithmic breakthroughs.

In addition to mining the endophytic bacteria contained in the transcriptome data, the new analysis strategy can simultaneously obtain species and abundance information, including plants, fungi, protozoa, and a small number of viruses. Compared to the amplicon sequencing method, the relative content between plants and microorganisms can be also obtained with the new analysis strategy. In this study, the content of plant rDNA in random samples was all above 99%, and the content of microbial rDNA ranged from 0.05% to 0.97%. The ability to obtain the relative content between plant and microbial genes was also one of the advantages of the new strategy. With the new strategy combined with Pavian, whether or not the inoculum exists in the plant tissue can be visualized. For example, after the inoculation of maize roots with R. irregularis, it could be detected that the fungi colonized inside the roots of maize, while it was hardly detected in the control. Researchers previously used transcriptome sequencing to study the role of maize LncRNAs in maize–AM fungal interactions, but the symbiosis after inoculation with R. irregularis was not shown in the study [44], while content changes of the fungi can be obtained using the transcriptome data stored at NCBI with the new strategy in this study. Similarly, information can be obtained from the transcriptome data of plants inoculated with other probiotics or pathogens. With the mining and content analysis of the marker microorganisms, the existence of the inoculated microorganisms in the corresponding tissues of the plant was also confirmed.

In the correlation analysis of replicate data, the new analysis strategy also showed that the composition and abundance of endophytic microorganisms obtained in different biological replicates are highly correlated, with a correlation coefficient between 0.87 and 1, which indicated that the results from the new strategy have good stability and consistency. In addition, when the amplicon was used to analyze the endophytic microorganisms of plants, it was necessary to extract the total DNA, then perform PCR amplification and sequencing, which required additional experimental costs. While the gene expression changes in plants with the transcriptome data were studied, the composition information of endophytic microorganisms in plants also could be analyzed with the new strategy at the same time, which effectively reduce research costs and improve data utilization. It was important to note that, in the operation process, surface microorganisms should be removed carefully in the processing of plant materials, and environmental microbial contamination should be avoided as much as possible during the sequencing process. Otherwise, the results will contain too much external microbial information, which will affect the reliability of the results.

The endophytic microbiome information obtained from the samples in this study can uncover the relative content between plants and microorganisms, and between different types of microorganisms. The microbial information included not only bacteria and fungi, but also viruses and protozoa. Generally, the relative content was bacteria > fungi > protozoa > viruses, but in maize kernels and roots, the relative content of fungi exceeded that of bacteria. In general, numerous studies have shown that bacteria are the predominant of all microorganisms [45,46,47], but the results of the new analysis strategy showed the composition of various types of microorganisms, from which it can be seen that eukaryotic microorganisms, including fungi and protozoa, sometimes dominate in specific tissues. In addition, protozoa are generally not a hot spot for research on plant endophytic microorganisms; however, in this study, we note that the relative content of protozoa should not be neglected, as they may also play an important role in promoting plant growth and other aspects. The relationship between microbial data and plant yield under different fertilization conditions (conventional, organic, and xylem bio-organic fertilizers) was examined [48], and it was found that protozoa are positively correlated with plant yield and the density of potential plant beneficial microorganisms. Protozoa can positively influence plant growth through interactions with beneficial plant microorganisms. The new analysis strategy in this study provides a new technical means for studying the interrelationships among plant microorganisms. It is very noteworthy that the microbial information obtained by the new analysis strategy belongs to living organisms, and the new analysis strategy should be also applicable to animal transcriptomes.

## 4. Materials and Methods

### 4.1. Plant Materials and Datasets

Maize kernels, maize leaves, tobacco roots, and tobacco stems were all provided by the laboratory, and Osmanthus leaves and Pittosporum leaves were randomly collected at Anhui agricultural University, China. All samples were soaked in 70% alcohol for 5 min and in 2% sodium hypochlorite solution for 3 min, rinsed with sterile water 5 to 7 times for disinfection, then immediately frozen in liquid nitrogen, and stored at −80 °C to use. All samples were divided into two parts, one for amplicon sequencing (BioProject: PRJNA879263) and the other for transcriptome sequencing (BioProject: PRJNA879262). Transcriptome data of some plants inoculated with pathogens or symbionts, including common bean inoculated with Xanthomonas (BioProject: PRJNA648388), maize root inoculated with AM fungus (BioProject: PRJNA553580), maize seedlings inoculated with Ustilago maydis (BioProject: PRJNA721951), phaseolus vulgaris inoculated with Rhizobium tropici (BioProject: PRJNA482464), were downloaded in NCBI.

### 4.2. Transcriptome Sequencing

Total RNA from the samples was extracted using the RNAiso kit. The RNA quality was analyzed by measuring the absorbance at 260 nm/280 nm (A260/A280). Sequencing libraries were constructed using a cDNA Synthesis kit, and sequencing was completed by BGI with BGISEQ platform (Shengzhen, China).

### 4.3. DNA Extraction and Amplicon Sequencing

The genome of the treated samples was extracted by the modified CTAB method, and the DNA quality was checked by 1% agarose gel electrophoresis. Using the extracted DNA as templates, the targeted V3-V4 region of the 16S rDNA was amplified by PCR reactions. Amplicon sequencing was performed by BGI. Amplicon sequences were analyzed using the EasyAmplicon process and spliced using FLASH software [49]. OTU clustering was performed using VSEARCH software, and chimeras in the sequences were detected and removed during the clustering process [50]. Representative sequences for each OTU were selected using QIIME2 software [30], and all representative sequences were compared and annotated with the RDP database.

### 4.4. Construction of the New Analysis Strategy

The database in this study is a self-built database, downloaded from NCBI, including genomes of bacteria (172,595), archaea (964), fungi (300), humans, protozoa (94) and virus (9362), plasmid sequences (3137) and nt library. Transcriptome data were assessed by fastqc software and cleaned by Trimmomatic (v0.33) with default parameters to obtain high-quality sequences, including removing linker and primer sequences, low quality start sequences and bases. Then the plant transcriptome data were split into two files with SortMeRNA software: one contained rDNA, and the other contained coding genes mostly. Files containing coding genes are generally used for transcriptome analysis; files containing rDNA were processed into new rDNA files with SortMeRNA software, which can be used directly for microbiome analysis (Figure 1).

The obtained rDNA sequence file contained the rDNA of the plant and the rDNA sequence of the microorganism. Kraken2, a metagenome analysis tool, was used to parse the rDNA sequence file into species composition information, including plants and microorganisms. Combined with the Bracken software, based on the Bayesian algorithm, which came with the Kraken2 software, the annotation and abundance information of endophytic microorganisms can be performed. Then the abundance of species in each sample, at the taxonomic levels of domain, kingdom, phylum, class, order, family, genus, and species, can be counted.

## 5. Conclusions

In this study, a new analysis strategy was developed to obtain endophytic microbiome information from plant transcriptome data. The new analysis strategy can obtain information on the composition and abundance of endophytic microbial communities, including archaea, bacteria, fungi, viruses, and protozoa, from the transcriptome sequencing data of plants directly. The relative content between plants and endophytic microorganisms, and between different types of endophytic microorganisms also can be obtained. The new analysis strategy can not only detect the content changes of endogenous microorganisms accurately, but also has strong data stability and consistency, while also effectively reducing research costs and improving data utilization.

## Figures and Tables

**Table 1:** Comparison of plant and microbial gene content in samples.

Sample	Tissue	Plant Reads (%)	Microbial Reads (%)
*O. fragrans*	leaf	96.63	3.37
*Pittosporaceae*	leaf	99.18	0.82
*N. tabacum*	stem	99.24	0.76
*N. tabacum*	root	74.99	25.01
*Zea mays*	kernel	98.73	1.27
*Zea mays*	leaf	99.01	0.99

**Table 2:** Content statistics of different microorganisms in samples.

Sample	Tissue	Bacterial Reads (%)	Viral Reads (%)	Fungal Reads (%)	Protozoan Reads (%)
*O. fragrans*	leaf	16.51	0.05	79.26	4.19
*Pittosporaceae*	leaf	27.94	0.26	69.00	2.79
*N. tabacum*	stem	46.34	0.00	41.23	12.43
*N. tabacum*	root	96.38	0.01	2.38	1.24
*Zea mays*	kernel	28.90	0.02	61.68	9.40
*Zea mays*	leaf	74.88	0.00	19.53	5.60

**Table 3:** Sequence information from 16S rDNA amplicon sequencing.

Sample	Tissue	Total Reads	Nonspecific Reads	Chloroplast Reads	Mitochondria Reads	Microbial Reads
*O. fragrans*	leaf	64,707	12	58,406	0	6289
*Pittosporaceae*	leaf	66,139	151	64,359	0	1629
*N. tabacum*	stem	66,852	316	59,594	0	6942
*N. tabacum*	root	60,093	59	24,970	0	35,064
*Zea mays*	kernel	64,699	521	46,588	0	17,590
*Zea mays*	leaf	67,381	249	66,983	0	149

## Data Availability

Not applicable.

## Supplementary Materials

The following supporting information can be downloaded at: https://www.mdpi.com/article/10.3390/ijms232214223/s1.
