# Supplementary material for: Establishment and Validation of a New Analysis Strategy for the Study of Plant Endophytic Microorganisms
Source: Int J Mol Sci. 2022 Nov 17;23(22):14223. doi: 10.3390/ijms232214223 (PMC9697482; doi:10.3390/ijms232214223)
Supplement: Supplementary file 1 [file ijms-23-14223-s001.zip › ijms-2005766-supplementary figure.pdf]

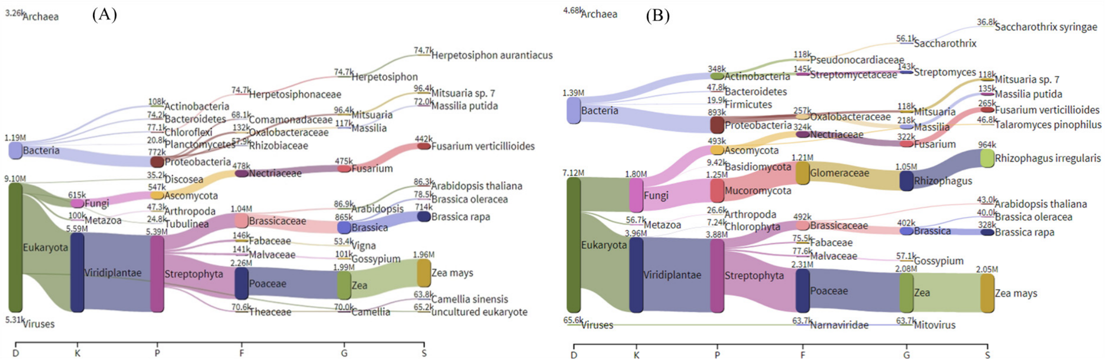

Figure S1. Sankey diagram of species composition of maize root. (A) Species composition before inoculated with AM fungus, AM fungus cannot be seen. (B) Species composition after inoculated with AM fungus, AM fungus (*Rhizophagus irregularis*) can be seen in the picture.

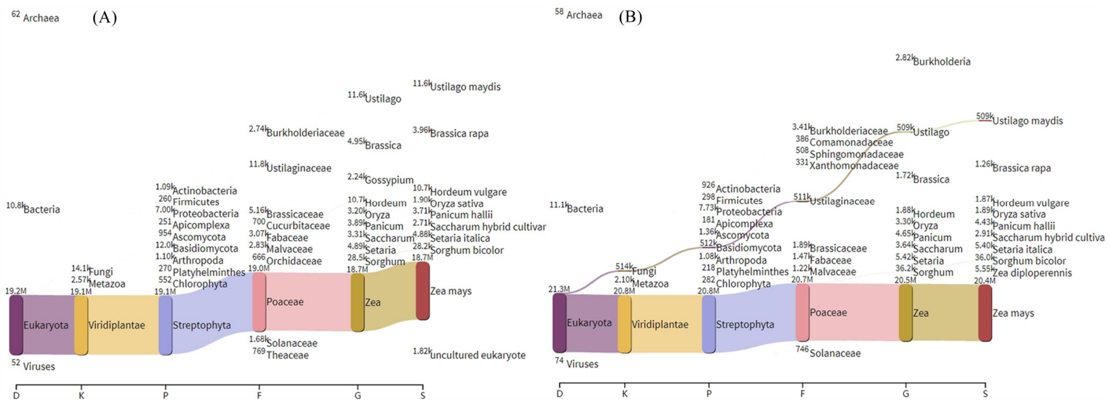

Figure S2. Sankey diagram of species composition of maize seedlings. (A) Species composition before inoculation with *U. maydis*, *U. maydis* cannot be seen. (B) Species composition after inoculation with *U. maydis*, *U. maydis* can be seen in the picture.
